# Active Management of Third Stage of Labor: Practice and Associated Factors among Obstetric Care Providers in North Wollo, Amhara Region, Ethiopia

**DOI:** 10.1155/2021/9207541

**Published:** 2021-12-31

**Authors:** Wondwosen Molla, Asresash Demissie, Marta Tessema

**Affiliations:** ^1^Department of Midwifery, College of Medicine and Health Science, Dilla University, Dilla, Ethiopia; ^2^School of Nursing, Faculty of Health Science, Institute of Health, Jimma University, Jimma, Ethiopia; ^3^School of Midwifery, Faculty of Health Science, Institute of Health, Jimma University, Jimma, Ethiopia

## Abstract

**Background:**

World Health Organization strongly recommends that every obstetrical provider at birth needs to have knowledge and skills on active management of the third stage of labor and use it routinely for all women. However, implementation of this lifesaver intervention by skilled birth attendants is questionable because 3% to 16.5% of women still experience postpartum hemorrhage. Even though coverage of giving births at health facilities in Ethiopia increases, postpartum hemorrhage accounts for 12.2% of all maternal deaths occurring in the country. Lack of the necessary skills of birth attendants is a major contributor to these adverse birth outcomes.

**Objectives:**

This study aimed to assess the active management of the third stage of labor practice and associated factors among obstetric care providers.

**Methods:**

An institution-based cross-sectional study design was applied from March 15 to April 15, 2020. Multistage sampling techniques were used to get 254 participants, and data were collected using self-administered structured questionnaires and an observation checklist. Data were entered into EpiData version 3.1 and exported to Statistical Package for the Social Sciences (SPSS) version 23.0 for analyses. The multivariable logistic regression model was used at 95% confidence interval with *P* value <0.05. Among the 232 providers participating in the study, only 75 (32.3%) of respondents had a good practice. The practice of the provider was significantly associated with work experience (adjusted odd ratio 0.206 (95% confidence interval, 0.06–0.63)), knowledge (adjusted odd ratio (2.98 (95% confidence interval, 1.45–6.14)), the presence of assistance (adjusted odd ratio 2.04 (95% confidence interval, 1.06–3.93)), and time of uterotonic drug preparation (adjusted odd ratio 4.69 (95% confidence interval, 2.31–9.53)).

**Conclusion:**

Only one-third of obstetric care providers had good practice during active management of third stage of labor. Practice was significantly associated with work experience, knowledge, the presence of assistance during third-stage management, and time of uterotonic drug preparation. Consistent and sustainable on job training and clinical audit should be applied in all facilities with regular supportive supervision and monitoring. Furthermore, team work and adequate preparation should be done to facilitate the management of active third stage of labor.

## 1. Introduction

Active management of the third stage of labor (AMTSL) is a combination of intervention performed by skilled birth attendant designed to facilitate the delivery of the placenta by increasing uterine contraction during the third stage of labor and also used to prevent postpartum hemorrhage (PPH) by averting uterine atony [1, 2].

According to FIGO-ICM and WHO, the usual components of AMTSL are the use of uterotonic agent in the first steps, preferably IM 10 IU oxytocin immediately within 1 min of delivery after ruling out the possibility of second baby to all births. Then, applying controlled cord traction (CCT) with the clamping of the cord within 1–3 minutes after birth is the second step for delivery of the placenta [3, 4].

The global target for ending preventable maternal mortality by 2030 recommends that every country should reduce its MMR by at least two-thirds from the 2016 baseline, and no country should have an MMR higher than 140 deaths per 100,000 live births (twice the global target) [5, 6]. In Ethiopia, the maternal mortality ratio was 412/100.000 live births in 2016 [7]. This number showed that MMR in our country is six times higher than the baseline, 140 deaths/100,000 live births [8].

Maternal mortality has sharply decreased by 43% worldwide [9, 10]. AMTSL plays an important role in preventing 27% of maternal deaths, 60% of PPH, and the use of blood transfusions [11, 12]. However, about 3% to 16.5% of women still experience PPH and require treatment [13–15]. Every pregnant woman may face life-threatening blood loss during delivery. FIGO-ICM strongly recommends that every obstetrical provider at birth needs to have knowledge, skills, and critical judgment to carry out AMTSL [1, 3].

PPH is one of the leading causes of maternal death worldwide. It occurs in 25% of all maternal deaths worldwide [4, 5], 34% in Africa [16], and 12.2% in Ethiopia [17], especially in settings where there are no birth attendants or where they lack the necessary skilled birth attendants and equipment to prevent and manage PPH and shock [12]. The majority of these deaths are preventable by adopting simple, effective, and safe strategies such as AMTSL [9, 16, 18].

Routine use of AMTSL by skilled birth attendants at health facilities for all vaginal singleton birth is recommended by the IFGO-ICM and WHO [3, 19]. All laboring women are at risk for PPH, so all obstetric care providers should have knowledge and skills regarding AMTSL intervention to prevent PPH [20].

The implementation of AMTSL intervention by skilled birth attendants is questionable because the incidence of PPH keeps rising [4, 13, 21]. However, studies have identified a gap in the use of AMTSL. In a global survey, it was found that only 16 (43%) of 37 investigated countries included the administration of a uterotonic/AMTSL in their national health management information systems [22]. A study conducted in seven Sub-Saharan countries reported that the AMTSL was only implemented correctly in 0.5–32% of the observed deliveries [23] whereas only 47% in Ethiopia [24].

## 2. Materials and Methods

### 2.1. Study Design and Setting

An institutional-based cross-sectional study design was conducted from March 15 to April 15, 2020, at governmental health facilities of Northeastern, Ethiopia.

### 2.2. Population

#### 2.2.1. Source Populations

The study included all obstetric care providers working in the maternity unit at North Wollo Health Facilities.

#### 2.2.2. Study Populations

The study included all obstetric care providers working at selected health facilities.

### 2.3. Inclusion and Exclusion Criteria

#### 2.3.1. Inclusion Criteria

All obstetric care providers working in the maternity unit were included in this study.

#### 2.3.2. Exclusion Criteria

Obstetric care providers who were not volunteers and those on annual leaves were excluded from the study.

### 2.4. Sample Size Determination

For two outcome variables, the sample size was determined using single population proportion formula by considering the following assumptions:

(1) $$\begin{array}{c} n=\frac{{\left(Z\_\alpha /2\right)}^{2}*P\left(1-P\right)}{d^{2}}, \end{array}$$

where *n* = sample size required for the study and *p* = the proportion of practice (32.8%) on AMTSL in Sidama Zone, South Ethiopia [14]. *Z* = Z*α*/2 = 1.96, corresponding to a 95% confidence level. *d* = the margin of error = 0.05.

For practice,

(2) $$\begin{array}{c} n=\frac{{\left(1.96\right)}^{2}\;*0.328\left(0.672\right)}{0.05^{2}}, \end{array}$$

where *n* = 338.7 approximately 339.

However, only 268 obstetric care providers were working at the selected health facilities. Because the population is less than 10,000, the study considered correction formula by taken 361 to get the maximum sample size:

(3) $$\begin{array}{c} nf=\frac{ni}{1+\left(ni/N\right)},\\ nf=\frac{361}{1+\left(361/268\right)},\\ n=153.8∼154. \end{array}$$

Since multistage sampling technique was used, the sample size was multiplied by the design effect of **1.5**:

(4) $$\begin{array}{c} n=1.5*154=231. \end{array}$$

For possible none response rate, the final sample size was increased by 10% to

(5) $$\begin{array}{c} n=254. \end{array}$$

The sample size for associated factors was determined using Epi Info 7 as shown in Table 1.

Therefore, the largest sample size was (*n* = 254).

### 2.5. Sampling Techniques

**Figure 1:**
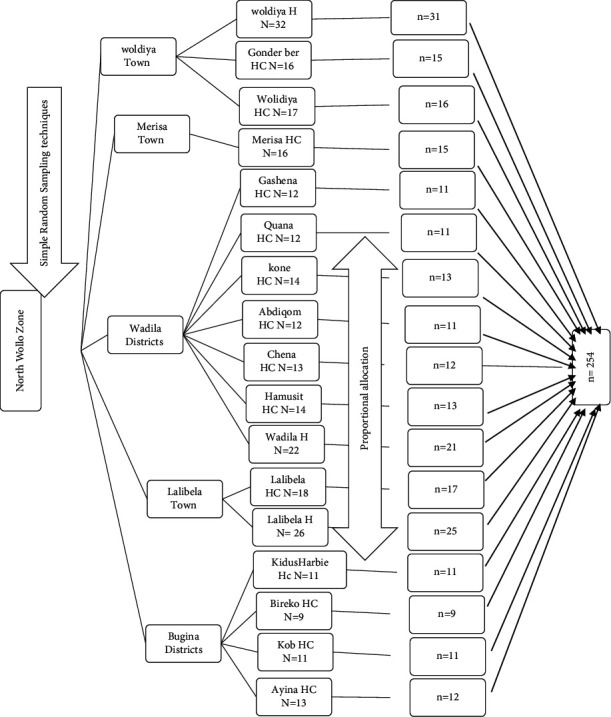
Schematic presentation of sampling procedure of the study. HC: health center, H: hospital, N: total number, n: sample.

Multistage sampling technique was used. Initially, out of 14 districts of the zone, five were selected using simple random sampling techniques (lottery methods): Lalibela districts, Wolidya districts, Mersa town, Wadila woreda, and Bugina woreda. There were 17 health facilities at the selected districts with a total of 268 obstetrics care providers. All the health institutions were included in this study. Then the final sample size was allocated proportionally for each health facility based on their number of obstetrical care providers. Respondents were selected by simple random sampling technique using the list of the professionals working in the delivery ward from the human resource management as a sampling frame, as shown in Figure 1.

### 2.6. Data Collection Tool and Data Quality Assurances

A standard questionnaire was adapted from the ICM and FIGO guidelines (1). It was prepared in English. Self-administered structured questionnaires and observation checks lists were used separately. Self-administered structured questionnaires have four parts: sociodemographic characteristics (9 items), training factors (7 items), health facility factors (7 items), and knowledge assessment (11 items). Moreover, the observation checklist part has 16 items. The level of knowledge was determined using 11 questions. Participants who scored below the median were considered to have poor knowledge. In contrast, participants who scored equal or above the median score were considered to have good knowledge. The level of practice was measured based on a series of 16 steps. Good practice: those who followed 16 steps of the checklist correctly while conducting AMTSL. Poor practice**:** those who did not follow at least one step of the checklist correctly.

A pretest was conducted on 5% of the sample size other than the study area. To minimize the effect of personal and professional relationships, observers were selected from outside of the study facilities. Cronbach's alpha was done to check the reliability. It was 0.84 for knowledge and 0.77 for practice. Eight data collectors and four supervisors who had Basic Emergency Obstetric and Neonatal Care (BEmONC) and Comprehensive Emergency Obstetric and Neonatal Care (CEmONC) training were recruited, and two days of training were given for both. Providers were not aware of the contents and items on the checklists. To minimize the effect of observation on the provider behavior (hawthorn effect), providers were assured the data collected were anonymous and individual performance would not be reported to their supervisors or shared publically (published reports only refer to aggregate data).

The birth attendants were observed while conducting the third stage of labor using the observational checklist. Then, self-administered structured questionnaires were distributed to respondents who participated in the observational part of the study. Data were collected and signed by supervisors after checking the filed questionnaire for any missing items and correctness. Besides, there was continuous follow-up and supervision by the principal investigator throughout the data collection period, and also necessary feedback was provided for supervisors and data collectors. All questionnaires and observation checklists were kept under lock and key for security and confidentiality of obtained information.

### 2.7. Data Analysis

Data were first checked manually for completeness, then coded and entered into EpiData version 3.1, and exported to SPSS version 23.0 for analyses. Exploratory data analysis was done to check missing values and outliers. The model fitness test was checked by Hosmer and Lemeshow test. The value was 0.869 for knowledge and 0.878 for practice. Binary and multivariable logistic regression analysis was done. Variables that had *P* value less than 0.25 in binary logistic regression were taken as a candidate for multivariable logistic regression. Finally, in multivariable logistic regression, variables with 95% CI at *P* value less than 0.05 were considered statistically significant.

## 3. Result and Discussion

### 3.1. Sociodemographic Characteristics of Respondents

From a total of 254 obstetric care providers included in the study, about 232 participated with an overall response rate of 91.3%. One hundred thirty-five (86%) participants were found in the age group of 25–30 years with a mean age of 28.7 years and a standard deviation (±SD) of 4.069, as shown in Table 2.

### 3.2. Health Institution and Training Information of Respondents

More than half (68.1%) of the participants were working at the health center, and almost all 229 (98.7%) and 226 (97.4%) of them had adequate oxytocin drugs and refrigerators in their workplace, respectively. About 35.3% of providers have in-service training as shown in Table 3.

### 3.3. Practice Obstetric Care Provider on AMTSL

**Figure 2:**
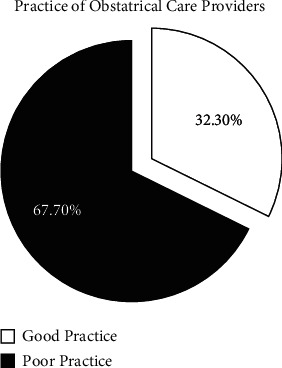
Practice of obstetric care provider on AMTSL at governmental health facilities in North Wollo, Amhara region, Ethiopia, 2020.

Only 32.3% of providers had a good practice on AMTSL in Figure 2. Moreover, the three components of AMTSL were more practiced among midwives compared to other professions as indicated in Table 4.

### 3.4. Knowledge of Obstetric Care Provider on AMTSL

Only 53.4% of respondents had good knowledge of AMTSL, as shown in Table 5. Furthermore, only 42.7% of respondents answered correctly about the administration of the uterotonic drug as a critical element of AMTSL. About 76.3% of providers responded that the time of cord clamping should be between 1 and 3 minutes.

### 3.5. Factors Associated with Practice of Obstetric Care Providers towards AMTSL

In multivariable logistic regression, the AMTSL practice of providers is significantly associated with work experience, knowledge on AMTSL, the presence of assistance during third-stage management, and time of uterotonic preparation, as shown in Table 6.

## 4. Discussion

### 4.1. Practice of Obstetric Care Provider on AMTSL

In this study, only 32.3% of providers followed AMTSL steps appropriately. It is almost in line with the study conducted in Sidama Zone (32.8%) [14], higher than the study done in Sudan (26.7%) [25] and (16.7%) Hawassa city [26], and lower than the studies conducted in Addis Ababa (47%) [24], Nigeria (78%) [27], and Netherlands (48%) [25]. The discrepancy might be due to knowledge gap, variation in study setting (in this study, both hospital and health center were included), and study participants (different disciplines included in this study).

Preloading uterotonic drugs before the third stage of labor, having assistant during third stage management, in-service training, work experience, and knowledge on AMTSL had a significant association with the provider's proper application of AMTSL. Considerable studies and different organizations indicate that team work and preparation to attend childbirth is mandatory to manage labor effectively and prevent unpredicted complications [1, 8]. In line with this, the present study also shows that providers who preloaded uterotonic drugs were 4.6 times more likely to practice AMTSL appropriately than those who did not preload. However, only 57.8% of care providers prepared uterotonic drugs before they started to attend labor. It is lower than the study done in Nepal, which was 99.3% [28]. The possible justification for this difference might be variation in study design (single-blind) and study setting. The study done in Nepal included only one training center hospital. This gives enhanced chances for the providers to access different on-the-job training that might have helped to update their knowledge. Moreover, providers working in the hospital mostly get experience from a gynecologist and other senior staff. Furthermore, providers who managed the third stage of labor with assistance were 2.0 times more likely to practice AMTSL appropriately compared to those who managed 3^rd^ stage of labor alone. However, still more than half of the providers (about 53.4%) managed the third stage of labor without the presence of assistance. This is higher than the study conducted in Nigeria, which is 23.7% [27]. The reason behind this result may be due to patient load and busy clinics. Nevertheless, due to the unpredicted nature of labor, having an assistant is a must.

Providers who had work experience <12 months were 79.4% less likely to practice AMTSL than those who had >36 months. This finding has a discrepancy with the study done in Indonesia, which indicates that participants who have less work experience practiced well. The variation might be due to the difference in study participants (research in Indonesia includes only midwives) and providers' burnout (due to workload and little benefits, some providers may develop burnout syndrome and do not do their work properly) [29]. On the contrary, Providers who received in-service training were 7.4 times more likely to practice AMTSL than those who did not. This finding is also supported by the study conducted in Tanzania [30]. In fact, providers who have been taken on in-service training might have better motivation and get practical training. Furthermore, those who had good knowledge of AMTSL were 2.9 times more likely to practice AMTSL than those who had poor knowledge. Similar to the studies conducted in Tanzania, Finfine area Special Zone, Nigeria, and Ghana [27, 30, 31]. The reason might be because knowledge brings a change in practice.

### 4.2. Limitation of the Study

Even if the different technique was carried out to minimize hawthorn effects, direct observations may change the provider's behaviors, so this could affect the results of the study.

## 5. Conclusions

Only one-third of obstetric care providers had good practice during active management of the third stage of labor. Practice is significantly associated with work experience, knowledge, the presence of assistance during third-stage management, and time of uterotonic drug preparation. The federal and regional health biro of the country should focus on updating the obstetric care provider's knowledge and skill by providing consistent and sustainable practice-based AMTSL training if a possible clinical audit should be applied in all facilities with regular supportive supervision and monitoring. Furthermore, the ministry of education should assess the quality of preservice training among health care providers.

## Figures and Tables

**Table 1:** Sample size determination using Epi Info for factors associated with practice.

Factors associated with practice	Assumption				Final sample size
	CI (%)	OR	Ratio	% of outcome in unexposed	
Access to reading materials	95	3.1	1 : 0	31.8	118
Knowledge	95	3.2	1 : 0	36.3	110
Qualification	95	5.5	1 : 0	60.0	82
Sex	95	5.6	1 : 0	45.4	62
Pre/in-service training	95	8.7	1 : 0	27.27	38

**Table 2:** Sociodemographic characteristic of participants, 2020.

Variable	Category	Frequency	Percent
Age	<25	49	21
	**25–30**	**135**	**58.2**
	31–35	34	14.8
	>35	14	6.0

Sex	Female	104	44.8
	**Male**	**128**	**55.2**

Marital status	**Married**	**121**	**52.2**
	Not married	100	43.1
	Divorced	8	3.4
	Widowed	3	1.3

Ethnicity	**Amhara**	**159**	**68.5**
	Oromo	22	9.5
	Tigre	47	20.3
	Others^*∗*^	4	1.7

Religion	**Orthodox**	**168**	**72.4**
	Muslim	47	20.3
	Protestant	17	7.3

Profession	General practitioner	42	18.1
	Health officer	32	13.8
	**Midwife**	**87**	**37.5**
	Nurse	71	30.6

Qualification	Diploma^*∗∗*^	54	23.3
	Advanced diploma^*∗∗∗*^	32	13.8
	**Degree** ^*∗∗∗∗*^	**146**	**62.9**

Year of graduation	<2000	1	4
	2000–2005	87	37.5
	**>2005**	**144**	**62.1**

Work experience	<12 months	41	17.7
	12–24 months	38	16.3
	25–36 months	48	20.7
	**>36 months**	**105**	**45.3**

Others^∗^ such as Afar, Gurage. Diploma^*∗∗*^: certificate given after two years of higher education training. Advanced diploma^*∗∗∗*^: type of diploma given after three years of higher education studies, just below the achievement of bachelor's degree. Degree^*∗∗∗∗*^: bachelor's degree.

**Table 3:** Health institution and training information of respondents, 2020.

Variable	Category	Frequency	Percent (%)
Facility	Hospital	74	31.9
	Health center	158	68.1

Reading material	Yes	116	50
	No	116	50

Favorable delivery ward	Yes	225	97
	No	7	3

Adequate uterotonic drug	Yes	229	98.7
	No	3	1.3

Refrigerator	Yes	226	97.4
	No	8	2.6

Training on AMTSL	Preservice	150	64.7
	In-service	82	35.3

**Table 4:** Practice of obstetric care provider on AMTSL, 2020.

Variables	Categories	Frequency	Percentage
Checked presence of another fetus	Yes	200	86.2
	No	32	13.8

Correct timing of administration uterotonic drug	Yes	192	82.8
	No	40	17.2

Types of uterotonic drugs given	Oxytocin	223	96.1
	Ergometrine	9	3.9

Correct dose of uterotonic drugs given	Yes	217	93.5
	No	15	6.5

Correct mode of administration of uterotonic drugs	Yes	225	97
	No	7	3

Correct timing of cord clamping	Yes	169	72.8
	No	63	27.2

Wait for uterine contraction 2-3 min to apply CCT	Yes	182	78.4
	No	50	21.6

Wait for gush of blood to apply cord control traction	Yes	201	86.6
	No	31	13.4

Placenta delivered before uterotonics administration	Yes	66	28.4
	No	166	71.6

CCT performed as protocol	Yes	178	76.7
	No	54	23.3

Placenta was supported by both hands	Yes	205	88.4
	No	27	11.6

Membrane extracted gently with lateral movement	Yes	205	88.4
	No	27	11.6

Uterine massage immediately after delivery of placenta	Yes	219	94.4
	No	13	5.6

Placenta assessed for completeness	Yes	220	94.8
	No	12	5.2

Uterine relaxation ensured	Yes	203	87.5
	No	29	12.5

Inform and demonstrate the mother massage uterus	Yes	205	88.4
	No	27	11.6

Overall practice	Good	75	32.3%
	Poor	157	67.7%

**Table 5:** Knowledge of obstetric care provider on AMTSL, 2010.

Variables	Categories			
	Yes	(%)	No	(%)
Know critical elements of the AMTSL	99	42.7	133	57.3
Know recommended immediate role of obstetric care providers after delivery of fetus	184	79.3	48	20.7
Know recommended first-line uterotonics drugs	215	92.7	17	7.3
Know recommended dose of oxytocin	201	86.6	31	13.4
Know recommended route of oxytocin	214	92.2	18	7.8
Know three main sequential components of AMTSL	202	87.1	30	12.9
Know time to administer uterotonics	201	86.6	31	13.4
Know recommended time to clamp the cord	177	76.3	55	23.7
Know the frequency of performing uterine massage over the first two hours	184	79.3	48	20.7
Know time of completing AMTSL	138	59.5	94	40.5
Know harmful practices when performing AMTSL	185	79.7	47	20.3
Adequate knowledge on AMTSL	124	53.4%	108	46.6%

**Table 6:** Bivariate and multivariable analysis, on factors associated with obstetrics care providers' practice on active management of third stage of labor, 2020.

Variables	Practice status		COR (95% CI)	AOR (95% CI)	*P* value
	Good	Poor			
Work experience					
**<12 months**	**5**	**36**	**0.200 (0.073–0.552)** *∗∗*	**0.206 (0.067–0.635)**	**0.01**
12–24 months	10	28	0.515 (0.227–1.169)	0.654 (0.251–1.706)	0.38
25–36 months	17	31	0.791 (0.390–1.605)	0.571 (0.252–1.293)	0.17
>36 months	43	62	1	1	
Manage 3^rd^ stage of labor with assistance					
**Yes**	**43**	**65**	**1.902 (1.090**–**3.320)***∗*	**2.045 (1.062–3.936)**	**0.02**
No	32	92	1	1	
Loading of uterotonic					
**Yes**	**59**	**75**	**4.032 (2.136–7.608)** *∗∗*	**4.695 (2.311–9.538)**	**0.01**
No	16	82	1	1	
Sex					
Male	50	78	2.026 (1.142–3.593)*∗*		
Female	25	79	1		
Profession					
GP	17	25	2.769 (1.184–6.473)*∗*		
HO	9	23	1.593 (0.606–4.191)		
Midwife	35	52	2.740 (1.327–5.657)*∗∗*		
Nurse	14	57	1		
Qualification					
Diploma	10	44	0.355 (0.165–.761)*∗∗*		
Advanced diploma	8	24	0.520 (0.219–1.238)		
Degree	57	89	1		
In-service training					
Yes	31	51	4.026 (1.720–9.424)*∗∗*		
No	53	150	1		
Knowledge of respondents on AMTSL					
Good knowledge	**57**	**67**	**4.254 (2.295–7.885)** *∗∗*	**2.986 (1.451–6.144)**	**0.01**
Poor knowledge	18	90	1	1	

Note: *∗P* < 0.05, *∗∗P* < 0.01, 1: reference group.

## Data Availability

The data used to support the finding of this study are available from the corresponding author upon request.

## Abbreviations

AMTSL: Active management of the third stage of labor
AOR: Adjusted odds ratio
BEmONC: Basic Emergency Obstetric and Neonatal Care
CCT: Controlled cord traction
CEmONC: Comprehensive emergency obstetric and neonatal care
CI: Confidence interval
COR: Crude odds ratio
EDHS: Ethiopian Demographic and Health Survey
EPIDATA: Epidemiological data version
EPIINFO: Epidemiological information
FIGO: Federation of International Gynecology and Obstetrics
ICM: International confederation of midwives
IM: Intramuscular
IU: International unit
IV: Intravenous
OR: Odds ratio
PPH: Postpartum hemorrhage
SBA: Skilled birth attendant
SDG: Sustainable development goals
SPSS: Statistical Package for Social Science
WHO: World Health Organization.
